# Trajectories of hypoxemia and pulmonary mechanics of COVID-19 ARDS in the NorthCARDS dataset

**DOI:** 10.1186/s12890-021-01732-y

**Published:** 2022-02-04

**Authors:** Daniel Jafari, Amir Gandomi, Alex Makhnevich, Michael Qiu, Daniel M. Rolston, Eric P. Gottesman, Adey Tsegaye, Paul H. Mayo, Molly E. Stewart, Meng Zhang, Negin Hajizadeh

**Affiliations:** 1 Donald and Barbara Zucker School of Medicine at Hofstra/Northwell, Hempstead, USA; 2 Frank G Zarb School of Business at Hofstra University, Hempstead, USA; 3 Center for Health Innovations and Outcomes Research, Feinstein Institute for Medical Research, Manhasset, USA

**Keywords:** ARDS, COVID-19, COVID, Respiratory system compliance, Pulmonary compliance

## Abstract

**Background:**

Understanding heterogeneity seen in patients with COVIDARDS and comparing to non-COVIDARDS may inform tailored treatments.

**Methods:**

A multidisciplinary team of frontline clinicians and data scientists worked to create the Northwell COVIDARDS dataset (NorthCARDS) leveraging over 11,542 COVID-19 hospital admissions. The data was then summarized to examine descriptive differences based on clinically meaningful categories of lung compliance, and to examine trends in oxygenation.

**Findings:**

Of the 1536 COVIDARDS patients in the NorthCARDS dataset, there were 531 (34.6%) who had very low lung compliance (< 20 ml/cmH_2_O), 970 (63.2%) with low-normal compliance (20–50 ml/cmH_2_O), and 35 (2.2%) with high lung compliance (> 50 ml/cmH_2_O). The very low compliance group had double the median time to intubation compared to the low-normal group (107.3 h (IQR 25.8, 239.2) vs. 39.5 h (IQR 5.4, 91.6)). Overall, 68.8% (n = 1057) of the patients died during hospitalization. In comparison to non-COVIDARDS reports, there were less patients in the high compliance category (2.2% vs. 12%, compliance ≥ 50 mL/cmH20), and more patients with P/F ≤ 150 (59.8% vs. 45.6%). There is a statistically significant correlation between compliance and P/F ratio. The Oxygenation Index is the highest in the very low compliance group (12.51, SD(6.15)), and lowest in high compliance group (8.78, SD(4.93)).

**Conclusions:**

The respiratory system compliance distribution of COVIDARDS is similar to non-COVIDARDS. In some patients, there may be a relation between time to intubation and duration of high levels of supplemental oxygen treatment on trajectory of lung compliance.

**Supplementary Information:**

The online version contains supplementary material available at 10.1186/s12890-021-01732-y.

## Introduction

A subset of patients with COVID-19 deteriorate despite supportive measures, requiring invasive mechanical ventilation for acute respiratory failure and acute respiratory distress syndrome (ARDS) [1]. Controversy has existed regarding the differences between COVID-related ARDS (COVIDARDS) versus other causes of ARDS [2–4]. For example, there appear to be a subset of patients with higher lung compliance despite profound hypoxemia [5]. The cohorts of COVIDARDS patients reported in the literature have been limited by sample size and have not included data on pulmonary mechanics. Herein, we describe the development of the NorthCARDS dataset which includes data on trajectories of illness, response to treatments and lung compliance for COVID-19 patients who were mechanically ventilated at one of the 12 acute care hospitals within the Northwell Health System. We then describe differences in demographics and treatments as well as trajectories of lung compliance and hypoxemia over the hospital course for patients with very low versus low-normal lung compliance. This work sets the stage for further data analytics among patients with COVIDARDS to better characterize phenogroups using readily available data elements from electronic health records.

## Methods

### Patients, study design and data collection

This is a retrospective study of intubated and mechanically ventilated patients with ARDS and COVID-19 who were admitted to one of 12 acute care hospitals within the Northwell Health System during the height of the pandemic in New York City (March 1–April 30, 2020). Northwell Health is the largest academic health system in New York, serving approximately 11 million people. Discharge disposition was available until Dec. 10th, 2020 for all patients in the cohort. Within the Northwell Health COVID-19 Research Consortium, the Northwell ARDS Research Collaborative was formed by a multidisciplinary group of clinical providers and research scientists (data scientists, biostatisticians and clinical trialists) to work on the creation of the NorthCARDS dataset.

All patients admitted to one of the 12 hospitals within the Northwell Health system during the time period of March 1 through April 30, 2020 were screened. Inclusion criteria were: Age > 18, COVID-19 polymerase chain reaction (PCR) test positive during the hospitalization, treatment with invasive mechanical ventilation, and PaO_2_: Fraction of Inspired Oxygen (FiO_2_) ratio (referred to as P/F) ≤ 300 while on Peak End Expiratory Pressure (PEEP) ≥ 5 during the hospital admission. The requirement for bilaterality of infiltrates as per the Berlin ARDS definition was confirmed based on a random sample of one hundred patients who were reviewed for radiographic findings of bilateral pulmonary involvement based on attending radiologist read of chest x-rays or CT scan. Surgical patients, identified by the presence of a brief operative note within 24 h of intubation time (Ti) were excluded unless the mechanical ventilation was for a post-operative state rather than for the procedure alone. Death was defined as in-hospital mortality during index admission, or within 30 days of hospital discharge if the patient was re-admitted.

Features relevant for understanding patients’ lung mechanics were extracted from the electronic health records of COVIDARDS patients. All available laboratory values, medications and oxygen supplementation concentration and mode as well as pulse oximetry results (SpO_2_) were recorded.

This study was considered by Northwell Health Institutional Review Board (IRB) as minimal risk using data collected for routine clinical practice, meeting the ethical standards framed in 1964 Declaration of Helsinki. The Northwell Health IRB waived the requirement for informed consent.

### Data definitions and assumptions

Several data assumptions needed to be made to structure the data. These included which fields contained the most valid and reliable data, and how best to handle missing data. For transparency, we outline assumptions for data structuring below and how we tested these assumptions. The Northwell ARDS Research Collaborative discussed each assumption to ensure that they reflected the clinical practice of providers caring for patients and their data entry into the electronic health record. Further details are provided in Additional file 1.

#### Oxygen delivery method, concentration, and degree of hypoxemia

The FiO_2_ delivered was calculated based on the following formula: for nasal cannula or non-rebreather face mask, each liter of oxygen flow added 0.04 to 0.21 (room air), with a maximum of 6 L per minute for nasal cannula and 15 L per minute for non-rebreather mask. In the instances where the delivery method was not recorded in the electronic medical record, the previous recorded method was presumed to have been continued, until change in flow rate or delivery method was noted. To be able to accurately map hypoxemia prior to intubation, we used both arterial blood gas data on partial pressure of oxygen (PaO_2_) and peripherally measured oxygen saturation (SpO_2_). We calculated SpO_2_:FiO_2_ ratios as well as PaO_2_:FiO_2_ ratios over time for each patient across their entire hospital stay. For separate analyses we converted SpO_2_:FiO_2_ to PaO_2_:FiO_2_ ratios (‘derived P/F’) to obtain an estimated trajectory of PaO_2_ over time [6] (derived P/F = ([SpO_2_:FiO_2_] − 64)/0.84). The assumption that derived P/F would have parallel trends compared to Arterial Blood Gas (ABG) based P/F was visually tested (Fig. 3). *Oxygenation Index* was calculated based on previously described formula [7] [FiO_2_ × mean airway pressure]/PaO_2_ *100, using the FiO_2_ post-intubation and ABG PaO_2_ in the first 24 h after Ti.

#### Respiratory system compliance

We used both static compliance (change in lung volume per unit change in pressure in the *absence of flow)* using the plateau pressure recorded in the electronic medical record, (Tidal Volume/[Plateau Pressure − PEEP]); and dynamic compliance using the Peak Inspiratory Pressure (PIP) (change in lung volume per unit change in pressure in the *presence of flow*), (Tidal Volume/[PIP − PEEP]) [8, 9] when patients were deeply sedated/paralyzed as described below. We only included values obtained at the time of full patient sedation, or the administration of intermittent bolus or continuous infusions of paralytics and if the difference in patient respiratory rate and set respiratory rate was < 2 breaths/minute (Additional file 1: Figure S6). We made the assumption that patients would not have a significant component of airway resistance for most COVID-19 respiratory failure patients in the early stage of disease (no more than a difference of 5–7 cmH2O between PIP and Plateau pressures), and that therefore this added pressure due to flow would have a minimal contribution to overall measured compliance. This assumption was tested by visualizing the difference between static and dynamic compliance seen over time (Additional file 1: Figure S3).

### Outcomes measured

In addition to establishing the NorthCARDS dataset, we sought to explore whether there were different phenogroups of COVIDARDS. The primary outcome was the number of patients in categories of lung compliance on the first day of ARDS, and the characteristics seen descriptively in each category. The secondary outcome was hospital mortality and discharge location. Ventilator parameters and respiratory mechanics were reported for each group of pre-defined compliance. Oxygenation, pulmonary mechanics, therapeutics, and hospital disposition data were available during the entire hospitalization course for all patients.

### Statistical analyses

Descriptive statistics included proportions for categorical variables and mean (standard deviation) and median (interquartile range) for continuous variables. We used the Welch’s t-test, proportions z-test, and/or Mood’s median test to compare very low and low-normal compliance categories. Significance of correlation between lung compliance and P/F ratio was tested using significance test for linear regression (Additional file 1: Figure S1). We examined the assumptions of this test using Harvey-Collier test (p = 0.817), Durbin–Watson statistic (1.946), Goldfeld-Quandt test (p = 0.991), and Jarque–Bera test of normality (p < 0.001). Violation of normality assumption was solved by log-transformation of the data (p = 0.084). We used two-sided p-value < 0.05 as the threshold of statistical significance. The data was analyzed using *Python 3.7* and several libraries including *pandas*, *numpy*, *matplotlib*, *scipy*, *nltk*, and *re*. Because the size of our dataset could lead to finding statistically significant associations without clinical significance, each outcome was reviewed for clinical significance by the clinicians in the Northwell ARDS Collaborative and results are discussed in the context of pathophysiological validity and other investigators’ results.

## Results

**Fig. 1:**
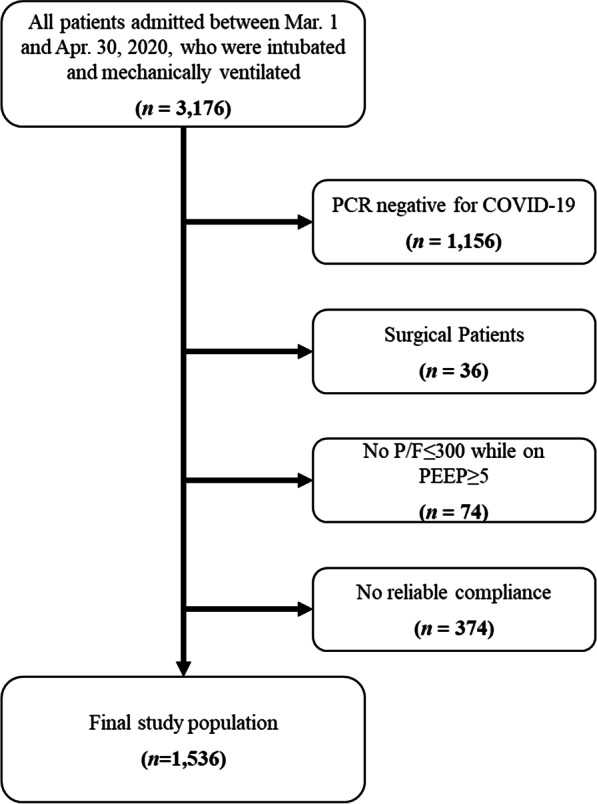
Flowchart of study population and exclusions

We identified 3176 patients who were admitted between March 1 and April 30, 2020 to one of the Northwell Health System hospitals, and who were mechanically ventilated. Of these, 2020 patients were COVID-19 PCR positive and 1536 met inclusion criteria with reliable lung compliance data (Fig. 1). Data for patients who were excluded are presented in Additional file 1 (Tables S1–S5). Discharge disposition for index hospitalization was available for all patients.

### Lung compliance categories

**Fig. 2:**
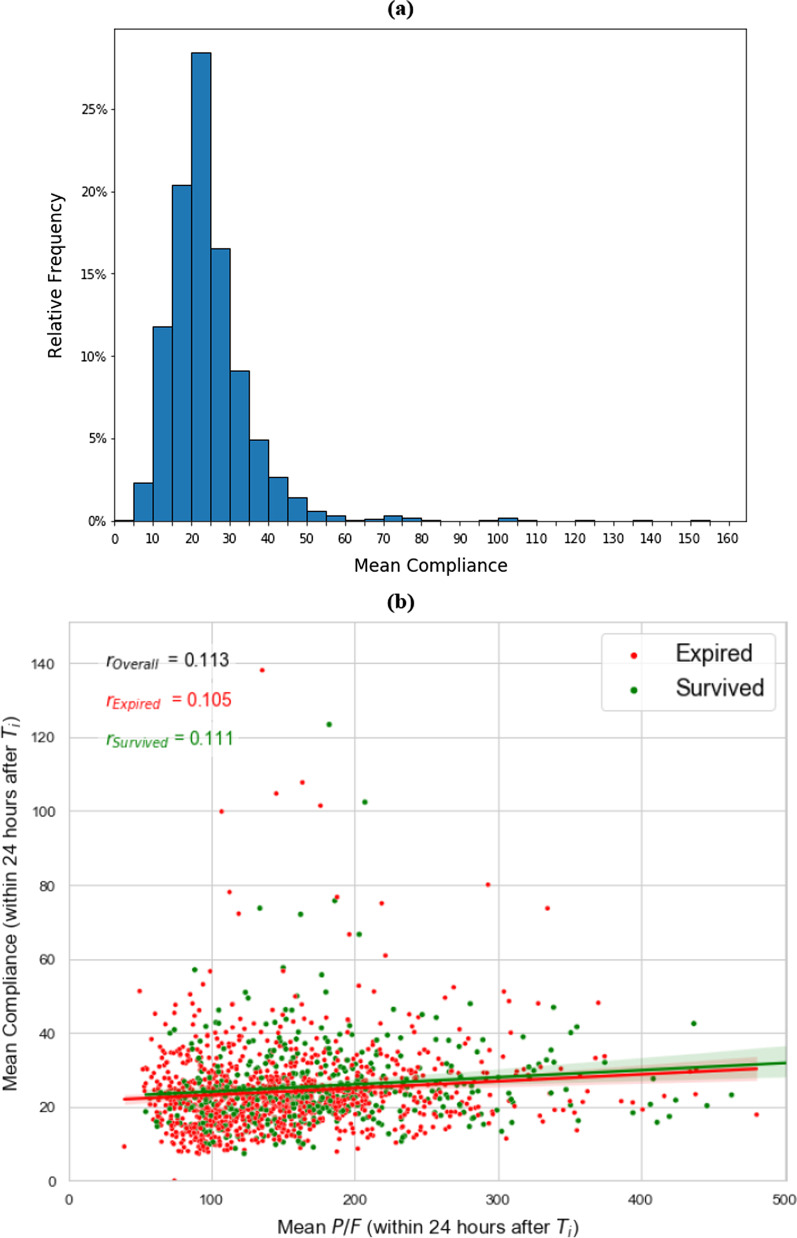
**a** Frequency of lung compliance seen in the first 24 h of mechanical ventilation for the entire cohort with reliable compliance data (n = 1536) by decile. **b** Correlation between Compliance and P/F from ABGs in the first 24 h of intubation

The average lung compliance in the first 24 h of mechanical ventilation for the whole cohort was 24.57 mL/cm H2O (SD 12.23). Frequencies per decile of compliance are presented in Fig. 2a. Based on clinical observations, the Northwell ARDS Collaborative chose to categorize the cohort into three categories: very low compliance (< 20 mL/cmH2O); low-normal (20–50 mL/cmH2O) and high (> 50 mL/cmH2O) measured by the dynamic compliance over the first 24 h of intubation in the setting of paralytics or deep sedation. There were 531 (34.6%) patients with very low compliance; 970 (63.2%) with low-normal compliance, and 35 (2.2%) with high compliance. Given the very small sample size in the higher compliance category, comparators of prevalence and exploratory statistical testing is limited to the very low versus low-normal compliance groups. The median difference between static and dynamic compliance overall was 6.41 mL/cmH2O (IQR 3.16, 11.43, n = 1053). For the very low compliance group median difference was 4.62 mL/cmH2O (IQR 2.08, 8.25, n = 428); and for the low-normal group 7.89 mL/cmH2O (IQR 4.17, 12.73, n = 596).

### COVIDARDS demographics

Patient demographics are detailed in Table 1. Overall, average age was 65 years, 32% were female, 35% were white, and the average Charlson comorbidity index was 4.9 (SD 3.2) (corresponding to a roughly 52% estimated 1-year survival) [10]. The Modified Early Warning Score (MEWS) was also high (4.1, SD 1.9) (corresponding to a roughly 12.7% chance of Intensive Care Unit (ICU) admission or death within 60 days) [11]. There was a greater percentage of females in the very low compliance category (43.7%, vs. 24.9%, low-normal; and 29.4%, high), and more were non-white/multi-racial. The most common comorbidity was hypertension (65.2%, n = 1001) and diabetes (43.4%, n = 666). The overall cohort included 16% (n = 244) with Body Mass Index (BMI) indicating extreme obesity (BMI > 40). Overall, three quarters of patients had preserved mentation in the 24 h preceding tracheal intubation, reflected in the fact that only 388 patients (25.3%) had an altered mental state. Notably, the highest proportion of patients with altered mental state was in the high compliance group (31%).

**Table 1:** Demographics stratified by high/normal/low compliance

	All patients with reliable compliance (n = 1536)	Patients with very low compliance (< 20 ml/cm H2O) (n = 531; 34.6%)	Patients with low–normal compliance (20–50 ml/cm H2O) (n = 970; 63.2%)	Patients with high compliance (> 50 ml/cm H2O) (n = 35; 2.2%)	p value (very low vs. low–normal compliance)
Age median (IQR)	65.0 (56.0,73.0)	64.0 (56.0,73.0)	65.0 (56.0,74.0)	69.0 (60.5,73.0)	0.042
Female n (%)	485 (31.6)	232 (43.7)	242 (24.9)	11 (31.4)	< 0.001
*Race*					
African American/Black n (%)	284 (18.5%)	111 (20.9%)	168 (17.3%)	5 (14.3%)	0.088
Asian n (%)	152 (9.9%)	68 (12.8%)	82 (8.5%)	2 (5.7%)	0.007
Other/Multiracial n (%)	481 (31.3%)	189 (35.6%)	274 (28.2%)	18 (51.4%)	0.003
White n (%)	537 (35.0%)	138 (26.0%)	391 (40.3%)	8 (22.9%)	< 0.001
Unknown n (%)	82 (5.3%)	25 (4.7%)	55 (5.7%)	2 (5.7%)	0.428
*Comorbidities*					
Charlson Index mean (SD)	4.9 (3.2)	4.7 (3.2)	5.0 (3.3)	4.4 (2.2)	0.178
MEWS on admission mean (SD)	4.1 (1.9)	4.2 (1.9)	4.1 (1.9)	3.9 (1.6)	0.224
BMI on admission mean (SD)	31.0 (7.1)	30.5 (7.4)	31.3 (7.0)	29.4 (4.3)	0.035
Chronic lung Disease n (%)	104 (6.8%)	35 (6.6%)	67 (6.9%)	2 (5.7%)	0.816
Diabetes n (%)	666 (43.4%)	232 (43.7%)	419 (43.2%)	15 (42.9%)	0.853
HTN n (%)	1001 (65.2%)	345 (65.0%)	638 (65.8%)	18 (51.4%)	0.755
CHF n (%)	115 (7.5%)	36 (6.8%)	77 (7.9%)	2 (5.7%)	0.416
CAD n (%)	204 (13.3%)	56 (10.5%)	144 (14.8%)	4 (11.4%)	0.019
CKD n (%)	226 (14.7%)	67 (12.6%)	156 (16.1%)	3 (8.6%)	0.071
ESRD n (%)	81 (5.3%)	25 (4.7%)	55 (5.7%)	1 (2.9%)	0.428
Positive Smoking n (%)	197 (12.8%)	64 (12.1%)	131 (13.5%)	2 (5.7%)	0.424
Malignancy n (%)	146 (9.5%)	50 (9.4%)	93 (9.6%)	3 (8.6%)	0.914
Altered mental status 24 h before intubation n (%)	388 (25.3%)	120 (22.6%)	257 (26.5%)	11 (31.4%)	0.096
*BMI categories*					
BMI < 18 underweight n (%)	9 (0.6%)	7 (1.3%)	2 (0.2%)	0 (0.0%)	0.008
BMI 18 to < 30 normal–overweight n (%)	733 (47.7%)	263 (49.5%)	453 (46.7%)	17 (48.6%)	0.294
BMI 30 to < 40 obese n (%)	550 (35.8%)	185 (34.8%)	350 (36.1%)	15 (42.9%)	0.631
BMI ≥ 40 extremely obese n (%)	244 (15.9%)	76 (14.3%)	165 (17.0%)	3 (8.6%)	0.173

### Interventions/treatments

Almost all patients (90%, n = 1376) received hydroxychloroquine, 62% (n = 958) received azithromycin, 83% (n = 1272) received steroids, 53% (n = 813) received paralytics, and 50% (n = 766) were proned. Prior to intubation, 281 patients (18.3%) were treated with awake-proning for various durations of time, Table 2 shows the rates of awake-prone in each compliance group. IL-1 and IL-6 inhibitors were given to 31% (n = 474) of the patients, while 7% received convalescent plasma (n = 109). During the first 48 h after intubation, 84% (n = 446) received at least one vasopressor in the very low compliance category, compared to 77.8% (n = 755) in the low-normal group. In general, few (48, or 3.1% of COVIDARDS) patients have received a CT angiography within the time frame of one day prior to 7 days after intubation to rule out pulmonary embolism as a contributing factor to hypoxemia. This was primarily due to the unsuitability of patients for transport to the CT suite due to hemodynamic instability as well as worsening hypoxia and desaturation with movement, in addition to concerns for infection control, given the limited knowledge regarding viral transmission at the time (Table 2).

**Table 2:** Interventions stratified by compliance groups

During the entire hospital stay	All patients with reliable compliance (n = 1536)	Patients with very low compliance (< 20 ml/cm H2O) (n = 531; 34.6%)	Patients with low–normal compliance (20–50 ml/cm H2O) (n = 970; 63.2%)	Patients with high compliance (> 50 ml/cm H2O) (n = 35; 2.2%)	p value (very low vs. low–normal compliance)
Steroids* n (%)	1272 (82.8%)	461 (86.8%)	781 (80.5%)	30 (85.7%)	0.002
Hydroxychloroquine n (%)	1376 (89.6%)	456 (85.9%)	888 (91.5%)	32 (91.4%)	< 0.001
Azithromycin n (%)	958 (62.4%)	268 (50.5%)	667 (68.8%)	23 (65.7%)	< 0.001
IL-1 or IL-6 inhibitor** n (%)	474 (30.9%)	188 (35.4%)	274 (28.2%)	12 (34.3%)	0.004
Remdesivir n (%)	11 (0.7%)	4 (0.8%)	7 (0.7%)	0 (0.0%)	0.945
Convalescent plasma n (%)	109 (7.1%)	46 (8.7%)	62 (6.4%)	1 (2.9%)	0.104
Proning n (%)	766 (49.9%)	315 (59.3%)	437 (45.1%)	14 (40.0%)	< 0.001
Proning before intubation n (%)	281 (18.3%)	163 (30.7%)	113 (11.6%)	5 (14.3%)	< 0.001
Paralytics n (%)***	813 (52.9%)	309 (58.2%)	489 (50.4%)	15 (42.9%)	0.004
Vasopressors (y/n) in first 48 h post intubation n (%)	1227 (79.9%)	446 (84.0%)	755 (77.8%)	26 (74.3%)	0.004
Inotropes (y/n) at any time count n (%)	65 (4.2%)	29 (5.5%)	36 (3.7%)	0 (0.0%)	0.111
Median hours from hospital presentation to intubation (IQR)	52.3 (7.8, 124.9)	107.3 (25.8, 239.2)	39.5 (5.4,91.6)	31.4 (1.6,79.0)	< 0.001
CT pulmonary angiogram (CTPA) performed within [-1 to 7 days] of intubation n (%)	48 (3.1%)	21 (4.0%)	26 (2.7%)	1 (2.9%)	0.175
Pulmonary embolus identified on CT n (% of patients with CTPA)	6 (12.5%)	2 (9.5%)	3 (11.5%)	1 (100.0%)	0.824
Pre-intubation O2 supplementation n (%) ****					
NRB	1186 (77.2%)	419 (78.9%)	739 (76.2%)	28 (80.0%)	0.23
NRB + NC	41 (2.7%)	18 (3.4%)	23 (2.4%)	0 (0.0%)	0.247
NC	80 (5.2%)	22 (4.1%)	56 (5.8%)	2 (5.7%)	0.174
HFNC	32 (2.1%)	12 (2.3%)	20 (2.1%)	0 (0.0%)	0.8
NIV (BiPAP/CPAP)	52 (3.4%)	23 (4.3%)	28 (2.9%)	1 (2.9%)	0.14
Venturi	16 (1.0%)	2 (0.4%)	14 (1.4%)	0 (0.0%)	0.054
Other*****	129 (8.4%)	35 (6.6%)	90 (9.3%)	4 (11.4%)	0.072

^*^Dexamethasone, hydrocortisone, methylprednisolone, prednisone, prednisolone

^**^Anikinra, Tocilizumab, Sarilumab

^***^Rocuronium, Vecuronium, Cisatracurium

^****^NRB = Nonrebreather Mask; NC- Nasal Canula; HFNC = High Flow Nasal Canula; NIV = Non-Invasive Mechanical Ventilation; Venturi = Venturi Mask

^*****^Other included room air; BVM; tracheostomy collar; simple face; bag mask; ambubag; T–piece; king airway;

### Time to intubation

On average, COVIDARDS patients were intubated within 52.3 h (IQR 7.8, 124.9) from the time of admission. Patients in the very low compliance group had the longest time between admission and intubation, 107.3 h (IQR 25.8, 239.2), compared to 39.5 h (IQR 5.4, 91.6) in the low-normal compliance group. Prior to intubation, 77% (n = 1186) of patients were receiving oxygen supplementation via non rebreather masks, with 2.1% (n = 33) on High Flow Nasal Cannula (HFNC), and 3.4% (n = 51) on Non Invasive Ventilation (NIV), which reflects infection control practices at the time discouraging NIV use (Table 2).

### P/F ratios and blood gas results

The average blood gas pH in the 24-h period before intubation was 7.30 (SD 0.12), and PaCO_2_ was 50.87 mmHg (SD 17.37) (Table 3). Patients in the very low lung compliance category had higher levels of PaCO_2_ and lower mean arterial pH. ABG was not performed in 71% cases during the 12 h prior to intubation. The overall mean derived P/F ratio in the 12 h prior to intubation was 93.39 (SD 83.21), which was lowest for those in the high compliance group (P/F 66, SD 33) (Table 3). When including PEEP in the calculation of P/F ratio, the P/FPEEP (PFP) [12] also appeared lowest for those in the highest compliance category. PFP ratio trends over time in the entire cohort is depicted in Additional file 1: Figure S7. Test of correlation shows a significant linear relationship between lung compliance and P/F ratio during the first 24 h after intubation for the entire cohort (p < 0.001), as well as in patients survived (p < 0.001), and expired (p < 0.001) (Fig. 2).

**Table 3:** Oxygenation trends and duration of ventilation by compliance group

	All patients with reliable compliance (n = 1536)	Patients with very low compliance (< 20 ml/cm H2O) (n = 531; 34.6%)	Patients with low–normal compliance (20–50 ml/cm H_2_O) (n = 970; 63.2%)	Patients with high compliance (> 50 ml/cm H_2_O) (n = 35; 2.2%)	p value (very low vs. low–normal compliance)
*P/F derived pre intubation (12 h mean)*					
Mean (SD)	93.39 (83.21)	81.56 (75.24)	100.55 (87.52)	74.37 (45.05)	< 0.001
Median (IQR)	60.00 (52.17,97.12)	58.29 (50.21,70.73)	61.84 (53.37, 117.66)	58.84 (52.57,69.56)	
n	1437	498	907	32	
*P/F from ABG pre intubation (12 h mean)*					
Mean (SD)	107.09 (86.74)	106.56 (97.26)	108.43 (81.68)	80.77 (22.83)	0.829
Median (IQR)	75.47 (61.73, 111.96)	71.60 (59.26, 109.20)	77.78 (63.76, 113.46)	75.31 (66.05,97.22)	
n	453	163	279	11	
*P/F derived post intubation, (12 h mean)*					
Mean (SD)	67.19 (38.42)	64.56 (36.30)	68.49 (39.42)	71.10 (40.67)	0.058
Median (IQR)	53.61 (40.99,80.13)	52.89 (40.18,74.66)	54.04 (41.40,83.63)	58.80 (40.36,87.91)	
n	1533	531	967	35	
*P/F from ABG post intubation (12 h mean)*					
Mean (SD)	151.00 (74.45)	139.88 (71.24)	156.83 (75.60)	160.74 (73.13)	< 0.001
Median (IQR)	133.88 (95.50, 188.41)	119.88 (92.00, 164.47)	143.42 (98.28, 198.19)	169.50 (95.00, 210.00)	
n	1476	515	928	33	
*First ABG P/F post intubation (within 4 h after Ti)*					
Mean (SD) n	142.91 (85.84)	129.70 (77.54)	150.22 (89.26)	158.76 (95.74)	< 0.001
Median (IQR)	119.00 (83.92, 179.00)	103.50 (81.75, 156.25)	125.00 (86.00, 194.50)	130.00 (84.29, 249.00)	
n	1116	408	683	25	
P/F derived gradient 24 h pre intubation*					
Mean (SD)	− 191.78 (1766.18)	− 213.06 (1230.52)	− 183.02 (2036.72)	− 94.58 (229.07)	0.778
Median (IQR)	− 7.37 (− 104.95,1.24)	− 4.79 (− 29.15,1.37)	− 9.87 (− 144.38,0.94)	− 10.68 (− 93.17,4.32)	
n	1236	440	769	27	
*Lactate pre intubation (24 h mean)*					
Mean (SD)	1.88 (2.58)	1.72 (1.74)	2.11 (3.35)	0.85 (0.31)	0.300
Median (IQR)	1.36 (1.00,1.85)	1.40 (1.00,1.81)	1.37 (0.97,1.90)	0.85 (0.65,1.05)	
n	200	107	89	4	
*Lactate post intubation (24 h mean)*					
Mean (SD)	1.68 (1.61)	1.84 (1.83)	1.52 (1.30)	0.73 (0.05)	0.193
Median (IQR)	1.30 (0.90,1.80)	1.40 (0.90,1.85)	1.30 (0.90,1.69)	0.72 (0.70,0.76)	
n	184	103	77	4	
*Number of hours with FiO2* ≥ *60% pre intubation*					
Mean (SD)	64.63 (100.48)	113.33 (135.88)	37.91 (58.14)	56.33 (110.16)	< 0.001
Median (IQR)	23.32 (2.33,82.38)	58.11 (8.50, 181.66)	16.08 (1.17,46.11)	15.50 (1.55,50.33)	
n	1473	514	927	32	
*Proportion of time on FiO2* ≥ *60% pre–intubation*					
Mean (SD)	62.5% (36.4%)	68.7% (34.5%)	58.7% (36.9%)	67.6% (37.2%)	< 0.001
Median (IQR)	74.4% (29.8%, 98.2%)	85.0% (42.3%, 98.6%)	65.7% (23.8%, 96.9%)	84.8% (39.4%, 100.0%)	
n	1403	499	873	31	
*Proportion of time on FiO2* ≥ *60% post–intubation*					
Mean (SD)	52.3% (34.6%)	54.9% (35.9%)	50.9% (33.9%)	52.3% (35.5%)	0.032
Median (IQR)	48.9% (18.8%, 88.9%)	55.0% (18.6%, 94.6%)	45.9% (19.0%, 84.9%)	52.7% (17.3%, 87.7%)	
n	1536	531	970	35	
Oxygenation Index**					
Mean (SD)	11.30 (5.92)	12.51 (6.15)	10.68 (5.69)	8.78 (4.93)	< 0.001
Median (IQR)	10.26 (6.95,14.16)	11.72 (8.32,15.52)	9.56 (6.61,13.51)	7.58 (6.25,10.68)	
n	1469	527	908	34	
PFP value***					
Mean (SD)	144.86 (102.90)	143.38 (93.86)	146.24 (108.67)	129.28 (63.09)	0.61
Median (IQR)	118.42 (84.00, 169.42)	118.33 (84.21, 168.28)	118.66 (83.92, 170.82)	114.24 (83.10, 168.11)	
n	1530	531	964	35	
*pH within –24 to* + *4 h from T*_*i*_					
Mean (SD)	7.30 (0.12)	7.26 (0.13)	7.32 (0.11)	7.33 (0.12)	< 0.001
Median (IQR)	7.32 (7.22,7.39)	7.28 (7.18,7.36)	7.34 (7.26,7.40)	7.35 (7.28,7.40)	
n	1119	410	686	23	
*PaCo2 within –24 to* + *4 h from T*_*i*_					
Mean (SD)	50.87 (17.37)	57.95 (19.08)	47.25 (15.21)	42.50 (10.17)	< 0.001
Median (IQR)	46.67 (39.00,59.00)	54.00 (44.00,70.00)	44.00 (37.60,53.00)	40.00 (35.50,45.00)	
n	1201	419	753	29	

Values are provided along with sample size (n) for patients with available data

*Additional file 1: Figure S1 shows the trend calculation method

**Oxygenation index = FiO_2_ × mean airway pressure]/PaO_2_ *100 (calculated using ABG PaO_2_ in the first 24 h after Ti)

***PFP Value = [P/(F × PEEP)] × 10 (calculated using ABG PaO_2_ in the first 24 h after Ti)

In the 12 h post intubation, the mean ABG P/F ratio was 151.00 (SD 74.45) for the overall group, and similar across groups (Additional file 1: Figure S2). Those in the very low compliance categories received higher FiO_2_ for longer periods of time prior to intubation (in the setting of also having longer average time to intubation). Trends of PaO2 and SpO2 immediately prior to intubation and 24 h after is available in Additional file 1: Figure S5. Prior to intubation, the group with normal to high compliance were exposed to FiO_2_ ≥ 60% for 56.33 h (IQR 1.55, 50.33) compared to 37.91 h (IQR 1.17, 46.11) in the low-normal category (Table 3).

**Fig. 3:**
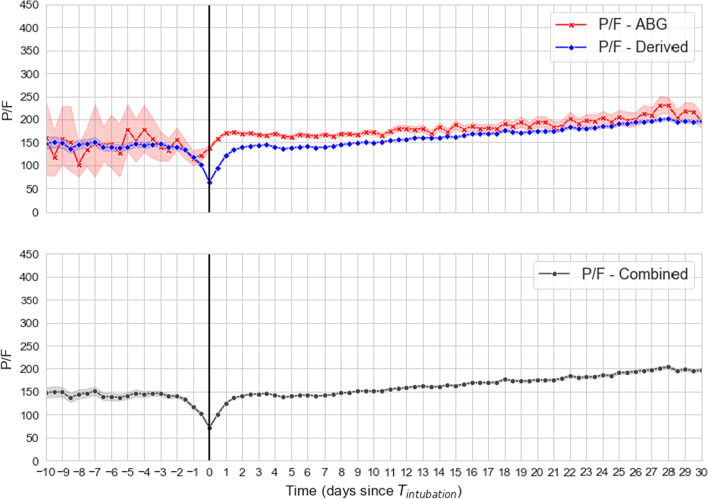
Trend over time in derived (from SpO2 from peripheral pulse oximetry) versus measured (from PaO2 in ABG) P/F ratio. The vertical black line denotes intubation time (Ti). Shaded areas indicate variability in measurements due to many missing measured PaO2 values relative to continually available SpO2 values. However, the direction of change over time is similar in derived and measured P/F values. The gap in derived and measured P/F during the first 24 h of mechanical ventilation likely represents a combination of the maximum SpO2 being 100% (as opposed to PaO2 which can be over 600) which sets an upper limit to the derived P/F from SpO2; and due to the shape of the oxygen dissociation curve wherein small changes in SpO2 correspond to larger changes in PaO2

**Fig. 4:**
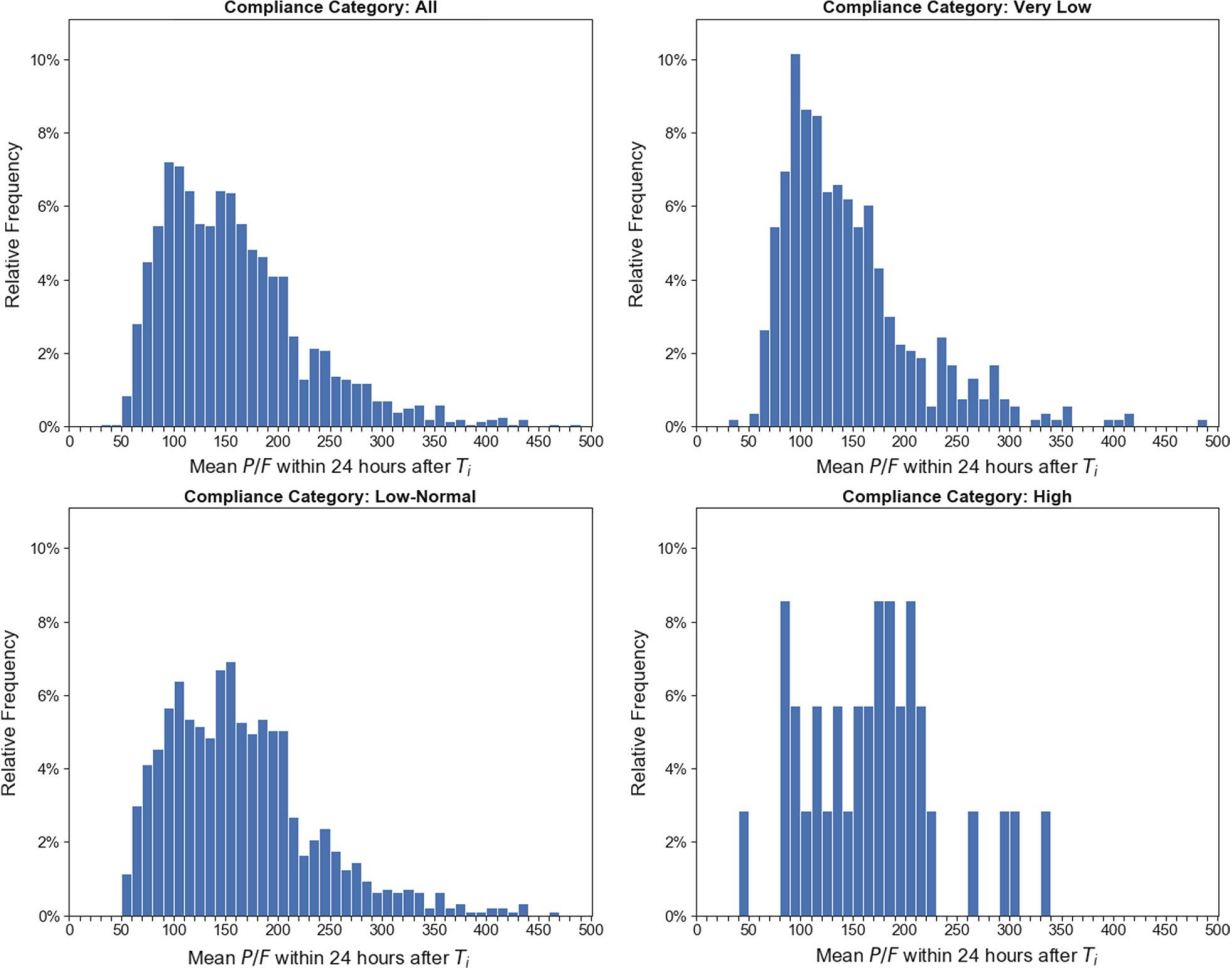
Frequency of ABG P/F by compliance category

The general trend of derived P/F ratios paralleled the ABG P/F ratios prior to intubation, although with high degree of variability among the ABG P/F ratios prior to intubation (wide 95% CI, shaded gray), due to many ABGs not being performed. Post-intubation, where many more ABGs were drawn, the two curves diverge for the first 48 h, and then trend together over time (Fig. 3). The P/F ratios distribution in each compliance group is depicted in Fig. 4.

### Oxygenation index (OI)

The mean OI for the entire cohort in the 24 h after intubation was 11.30 (5.92) and was slightly worse in the very low compliance group 12.29 (5.70) (Table 3).

### Duration of intubation

The average duration of intubation was 15.26 days (SD16.54). Among those who survived, median duration was 12.05 days (IQR 5.16, 29.13) and mean was 19.95 (STD 20.28) days. Among those who died, median duration was 9.04 days (IQR 4.10, 17.53) and mean was 13.07 (STD 14.02). It should be noted that the length of intubation for survivors is an underestimation due to the fact that 13.2% of survivors were discharged while still mechanically ventilated (Table 4).

**Table 4:** Mechanical ventilator obtained parameters (lung mechanics and ventilator settings)

	All patients with reliable compliance (n = 1536)	Patients with very low compliance (< 20 ml/cm H_2_O) (n = 531; 34.6%)	Patients with low–normal compliance (20–50 ml/cm H_2_O) (n = 970; 63.2%)	Patients with high compliance (> 50 ml/cm H_2_O) (n = 35; 2.2%)	p–value (very low vs. low–normal compliance)
*Compliance in the first 24 h of intubation*					
Mean (SD)	24.57 (12.23)	15.41 (3.26)	27.67 (6.49)	77.49 (30.27)	< 0.001
Median (IQR)	22.65 (17.97,28.24)	15.86 (13.26,18.26)	25.63 (22.74,30.95)	72.00 (54.36,89.90)	
n	1536	531	970	35	
*Mean PEEP (cm H2O) within 24 h of intubation*					
Mean (SD)	12.58 (3.75)	11.67 (3.63)	13.00 (3.68)	14.65 (4.41)	< 0.001
Median (IQR)	12.40 (10.00,15.00)	11.45 (9.35,14.27)	13.04 (10.00,15.22)	15.00 (10.88,18.31)	
n	1536	531	970	35	
*Mean Peak pressure (cm H2O) within 24 h of intubation*					
Mean (SD)	32.86 (6.56)	37.91 (6.18)	30.38 (4.89)	24.93 (5.29)	< 0.001
Median (IQR)	32.41 (28.52,36.59)	37.04 (34.08,41.65)	30.39 (27.09,33.55)	25.52 (22.78,27.16)	
n	1536	531	970	35	
*Ventilation duration (days)*					
Mean (SD)	15.26 (16.54)	15.93 (18.18)	14.81 (15.59)	17.65 (16.27)	0.211
Median (IQR)	9.77 (4.35,19.85)	10.06 (4.21,19.99)	9.69 (4.43,19.24)	9.56 (4.96,30.16)	
n	1536	531	970	35	
*Ventilation duration (days) among those who survived*					
Mean (SD)	19.95 (20.28)	23.00 (23.87)	18.64 (18.42)	15.88 (13.15)	0.030
Median (IQR)	12.05 (5.16,29.13)	13.44 (5.92,31.50)	11.95 (4.55,27.72)	10.62 (7.28,25.48)	
n	479	152	315	12	
*Ventilation duration (days) among those who died*					
Mean (SD)	13.07 (14.02)	13.02 (14.40)	12.90 (13.63)	18.57 (17.89)	0.902
Median (IQR)	9.04 (4.10,17.53)	8.48 (3.77,17.77)	9.24 (4.33,16.91)	8.71 (4.86,31.44)	
n	1057	379	655	23	
*Vt cc/Kg of IBW within 24 h of intubation*					
Mean (SD)	6.77 (1.16)	6.80 (1.23)	6.77 (1.14)	6.46 (0.82)	0.63
Median (IQR)	6.63 (6.05,7.35)	6.68 (6.05,7.38)	6.62 (6.06,7.35)	6.46 (6.01,6.91)	
n	1441	507	902	32	
*Set respiratory rate (per minute) within 24 h of intubation*					
Mean (SD)	24.23 (5.11)	26.36 (5.24)	23.15 (4.66)	21.95 (4.66)	< 0.001
Median (IQR)	24.09 (20.00,28.00)	26.67 (22.53,30.04)	23.48 (20.00,26.41)	21.73 (19.00,25.30)	
n	1536	531	970	35	
*Total respiratory rate within 24 h of intubation*					
Mean (SD)	25.72 (4.75)	27.64 (4.85)	24.72 (4.38)	24.30 (4.26)	< 0.001
Median (IQR)	25.69 (22.25,29.20)	28.00 (24.42,31.23)	24.76 (21.60,27.84)	24.00 (21.10,27.83)	
n	1536	531	970	35	
*Plateau pressure (cm H2O) within 24 h of intubation*					
Mean (SD)	28.37 (6.59)	31.93 (6.60)	26.05 (5.15)	20.68 (8.11)	< 0.001
Median (IQR)	28.00 (24.25,32.00)	31.25 (27.50,35.80)	26.00 (22.33,29.48)	20.17 (16.25,27.75)	
n	1049	429	602	18	
Driving pressure* (cm H20) within 24 h of intubation					
Mean (SD)	16.18 (6.44)	20.47 (6.82)	13.31 (3.93)	8.60 (5.32)	< 0.001
Median (IQR)	15.00 (12.00,19.06)	19.42 (16.00,24.08)	13.00 (10.67,15.50)	10.00 (4.62,11.33)	
n	1044	428	600	16	

^*^Driving pressures were reported only when plateau and PEEP was recorded at same time

### Lung mechanics and ventilator settings

**Fig. 5:**
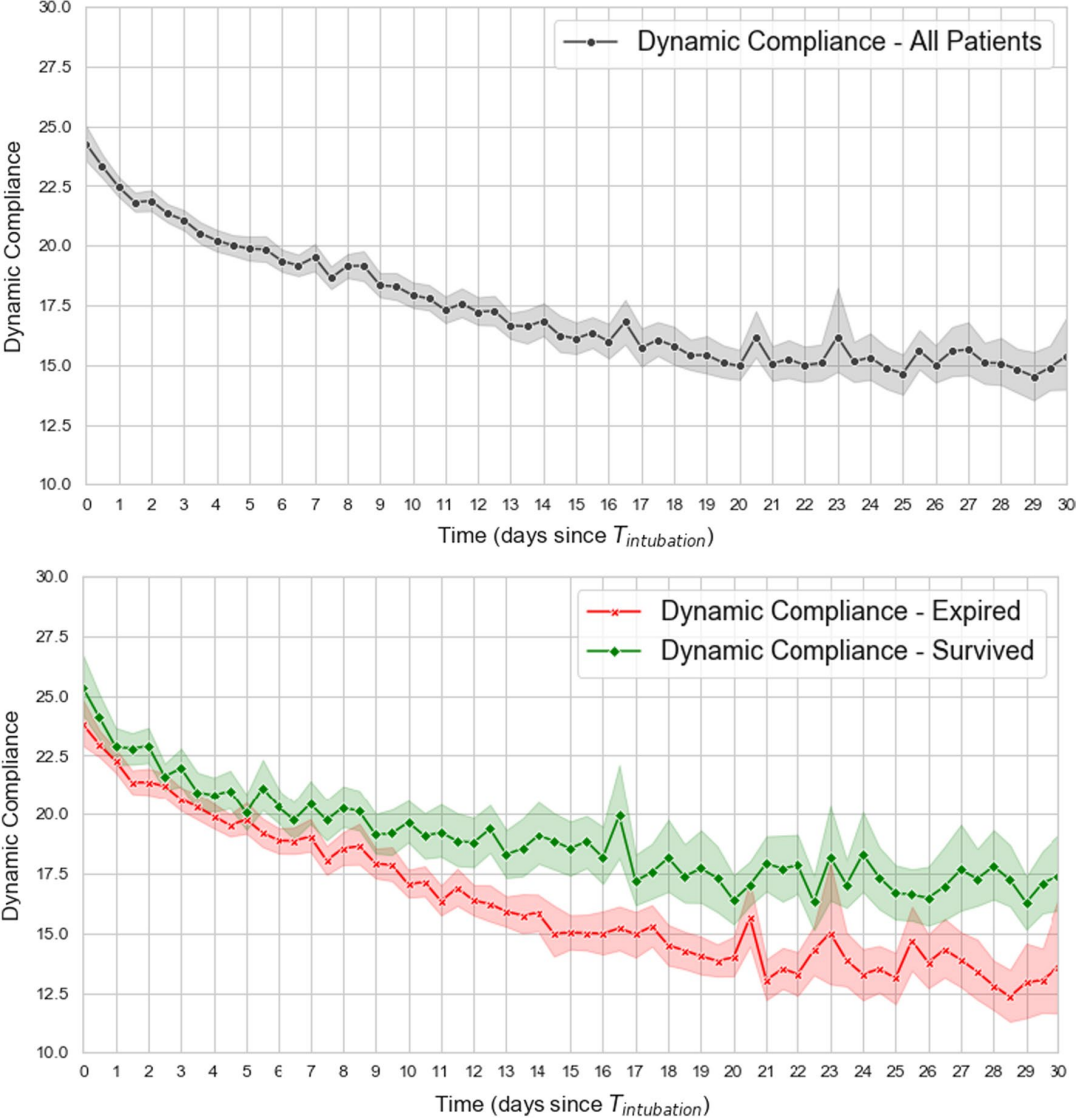
Trends in compliance for the total cohort [n = 1536] for the first 5 days post intubation (top), and 30 day trends of dynamic compliance between survivors and non–survivors (bottom) indicating decreasing compliance over time with a steeper decline among non–survivors. Trends per compliance category are presented in Additional file 1

Lung compliance for the whole cohort decreased over time, with a steeper trajectory among those who died (Fig. 5). This was seen more clearly in the low-normal compliance group and high compliance groups likely secondary to the ‘floor effect’ (very low compliance numbers starting at a very low value) (Additional file 1: Figure S4). On average, patients received 6.77 cc/kg (SD 1.16) of ideal body weight as the ventilator setting (Table 4). As expected, the very low lung compliance group had the highest average peak airway pressure, plateau pressure, and resulting driving pressures. The mean driving pressure for the whole cohort was 16.18 (SD 6.44), and 20.47 (SD 6.82) for the very low compliance group compared to 13.31 (3.93) for the low-normal compliance group.

### Proportion of deaths and discharge to home

Table 5 presents the disposition status of patients based on the index hospitalization which was available for all patients (unknown for one patient). Overall, of the 1536 patients, 68.8% (n = 1057) died during the index hospital stay. Of the 479 patients who survived to hospital discharge, 63 (13.2%) were discharged while still on a mechanical ventilator. Of those who survived, 56.8% (n = 272) were discharged home and the rest to rehabilitation or long-term care facilities. The very low compliance group had the highest mortality (71.4% versus 67.5%).

**Table 5:** Hospital mortality and discharge location stratified by compliance group

	All patients with reliable compliance (n = 1536)	Patients with very low compliance (< 20 ml/cm H_2_O) (n = 531; 34.6%)	Patients with low–normal compliance (20–50 ml/cm H_2_O) (n = 970; 63.2%)	Patients with high compliance (> 50 ml/cm H_2_O) (n = 35; 2.2%)	p value (very low vs. low–normal compliance)
Deceased % (n)	68.8% (1057)	71.4% (379)	67.5% (655)	65.7% (23)	0.124
Survived % (n)	31.2% (479)	28.6% (152)	32.5% (315)	34.3% (12)	0.124
Discharged while on mechanical ventilator, % of the survivors (n)	13.2% (63)	15.8% (24)	11.4% (36)	25.0% (3)	0.187
Discharged home, % of the survivors (n)	56.8% (272)	57.9% (88)	56.5% (178)	50.0% (6)	0.777
Discharged to another facility including acute care and longer-term rehabilitation, % of the survivors (n)	43.0% (206)	42.1% (64)	43.2% (136)	50.0% (6)	0.827

## Discussion

Patients with COVIDARDS in the NorthCARDS dataset had heterogeneous lung compliance, as measured in the first 24 h of intubation. Three observations were particularly notable and include the longer time to intubation for patients with very low lung compliance, the steeper trajectory of compliance decrease seen among those who died, and the severity of hypoxemia in those with high lung compliance. As others have noted, the course of COVID19 pneumonia and ARDS appears to start with a highly compliant lung but with profound hypoxemia [13, 14]. Therefore, it is possible that ARDS patients with low compliance detected at the time of intubation may well have started with a normal lung compliance with deterioration during the course of illness, in part due to the disease process itself, and possibly due to treatments administered. For example, it is possible that prolonged exposure to high concentrations of oxygen contributed to the low compliance seen once patients were intubated. This is suggested by the fact that the very low compliance group spent the greatest number of hours as well as proportion of time prior to intubation on FiO_2_ > 60%. High concentrations of oxygen have been demonstrated to cause lethal lung injury in animal models [15–17], and have been associated with increased mortality [18, 19] , severe lung injury, and pneumonia [20] in humans. A recent study linked hyperoxia to microbial dysbiosis in both the lung and gut microbiome which could contribute to the lung injury [21]. It is unclear whether earlier intubation, and/or lower oxygen saturation thresholds would have mitigated worsening of lung compliance. The recent ICU-ROX study did not find that conservative oxygen thresholds (SpO_2_ 90–97%) decreased ventilator days in intubated ICU patients [22], and the recent LOCO2 trial, conservative therapy (SpO_2_ 88–92%) was associated with increased mortality among intubated patients [23]. However, these results may not apply to non-intubated patients. Many COVID-19 patients who were maintained without intubation had uniquely preserved mentation despite very low SpO_2_ levels (likely due to right-shifted oxygen dissociation curves) and did not meet conventional thresholds for intubation. Alternatively, clinicians have posited that PSILI (patient self-induced lung injury) [24] due to extreme respiratory drives could exacerbate lung damage in COVID-19 disease. Prior to intubation patients were not receiving sedation and strong respiratory drives may have contributed to the lower lung compliance seen due to PSILI. Of course, these patients could have had very low compliance at the time of hospital presentation. In addition, the persistence of active disease itself could have led to progressively lower compliance due to persistent severe inflammation. A prospective study that includes a surrogate measure for compliance prior to intubation, ideally with serial measurements over time, and documentation of progression of ventilation and perfusion mismatch (including ultrasound or other radiography and dead space estimation) will help answer these questions. These investigations are relevant for ARDS in general and findings will have implications for the management of ARDS beyond COVID-19.

Degree of ventilation to perfusion (V/Q) mismatch and hypoxemia does not appear to correlate with lung compliance, which corresponds to what colleagues have found in the non-ARDS analyses [25]. Indeed, 42% of the cohort with high lung compliance in non-COVID ARDS patients had P/F levels under 150, which is similar to our findings in COVIDARDS. The extremely low P/F ratio, P/FP and high Oxygenation Index seen among patients in the high compliance group suggests ventilation perfusion mismatch which could be explained by the extensive micro-thrombi that have been reported, and the involvement of the vascular endothelium with impaired hypoxic pulmonary vasoconstriction [26]. Questions have been raised about whether COVIDARDS should be treated differently than non-COVIDARDS. The more relevant question seems to be whether ARDS management should be different for patients with different severity of lung compliance impairment and different degrees of ventilation and perfusion mismatch. Ongoing studies are exploring whether respiratory mechanics will change with the implementation of different treatment strategies. An index that takes into account oxygen impairment and compliance over time, pointing to predominance of dead space ventilation (thrombi) versus shunt physiology (alveolar and parenchymal pathology, and impaired vascular hypoxic vasoconstriction) may help clinicians tailor treatments for individual patients with ARDS.

Only 2.2% of patients were in the high compliance category (low elastance/ phenotype “L”) [27]. This is lower than the 12% reported in the recent secondary analysis of the LungSAFE data of non-COVID ARDS patients [25]. However, it is important to note that our description of compliance variability is limited to ARDS patients who are mechanically ventilated. Many patients who met ARDS criteria based on hypoxemia and bilaterality of infiltrates did not receive mechanical ventilation until several days after admission. This period was likely prolonged compared to other viral pneumonia causes of ARDS due to the relatively preserved mental status in COVID-19 patients despite profound hypoxemia. Comparisons between studies need to consider the timing of intubation relative to symptom onset, and different practice patterns regarding thresholds for intubation. Disparate outcomes reported internationally are likely explained in large part by different comorbidity burden, severity of hypoxemia on hospital presentation, and different practice patterns regarding timing of intubation.

The strengths of this study include being the largest sample of COVIDARDS patients in a single health system which has granular patient-level data regarding respiratory mechanics and oxygenation. We have described methods for leveraging real-world data to determine lung compliance data in the absence of patient effort which could either over- or under-estimate true pressures. Our large sample size allowed us to maintain 1536 patients in the dataset who had reliable data on pulmonary mechanics.

Limitations of the present study are inherent to the retrospective nature of this data extraction from the electronic health record. Of particular note is that many of these patients were cared for during March 2020, and at the time the provision of PEEP was limited to invasive mechanical ventilation due to infection control concerns. We are unable to ensure that there was no significant airway resistance contributing to the measurement of dynamic compliance, and to account for the contribution of abdominal pressures and chest wall stiffness. The small and consistent margin of difference between static and dynamic compliance seen suggests that airway resistance contributed minimally to measured dynamic airway pressures. We assumed that the difference between dynamic and static compliance would be < 10 mL/ cmH_2_O due to airway resistance not being commonly observed in the early stages of COVIDARDS. In non-COVID-19 related ARDS the mean difference between peak and plateau pressures has been found to be 6–7 cmH_2_O [28]. However, given that 50% (n = 804) of patients had a BMI of over 30, it is possible that chest wall compliance contributed to a decreased measured compliance in some patients. We did not exclude patients who may have had an additional component of cardiogenic pulmonary edema as a cause of ARDS as we did not have a valid measure of cause of pulmonary edema in the dataset. However, inotropic support was not a prevalent feature in these patients and severe hypoxemia due to cardiogenic pulmonary edema alone is less common in COVID-19. A further limitation is our inability to control for factors which influenced decisions about timing of intubation for COVID-19 patients, as protocols are difficult to establish for this disease which presents in many with discordant mental status for degree of hypoxemia. For example, those who were intubated earlier may have had altered mental status which could confound differences seen in mortality associated with lung compliance. Limits to resuscitation due to patient and family preference have also not been presented in this descriptive analysis. On the other hand, different reasons and thresholds for intubation can be leveraged to further define best management and is the subject of ongoing research.

In summary, we present the methods for establishing the NorthCARDS dataset of COVIDARDS patients, and the range of lung compliance and oxygen trajectories seen in these patients. These data will inform phenogrouping research to further understand COVIDARDS towards tailored approaches to treatment which maybe also be applicable to non-COVID-19 related ARDS.

## Conclusions

The respiratory system compliance distribution of COVIDARDS is largely similar to non-COVIDARDS, with most patients having low or very low lung compliance. Patients with high lung compliance had profound hypoxemia. In some patients, there may be a relation between time to intubation and duration of high levels of supplemental oxygen treatment on trajectory of lung compliance.

## Supplementary Information

**Additional file 1**. Data Assumptions and Structuring.

## Data Availability

The datasets used and/or analyzed during the current study are available from the corresponding author on reasonable request.

## Abbreviations

ARDS: Acute Respiratory Distress Syndrome
COVIDARDS: COVID related acute respiratory distress syndrome
PCR: Polymerase chain reaction
PaO2: Oxygen partial pressure
FiO2: Fraction of inspired oxygen
P/F ratio: PaO_2_/FiO_2_
CT: Computed tomography
Ti: Intubation time
SpO2: Oxygen pulseoximetry
IRB: Institutional Review Board
ABG: Arterial blood gas
PEEP: Peak end expiratory pressure
PIP: Peak inspiratory pressure
SD: Standard deviation
MEWS: Modified Early Warning Score
ICU: Intensive care unit
BMI: Body mass index
IQR: Interquartile range
NIV: Non invasive ventilation
HFNC: High flow nasal cannula
PaCO2: Carbon dioxide partial pressure
PFP: PEEP × P/F
OI: Oxygenation index
PSILI: Patient self inflicted lung injury
V/Q: Ventilation to perfusion
