# Supplementary material for: Trajectories of hypoxemia and pulmonary mechanics of COVID-19 ARDS in the NorthCARDS dataset
Source: BMC Pulm Med. 2022 Feb 4;22:51. doi: 10.1186/s12890-021-01732-y (PMC8814783; doi:10.1186/s12890-021-01732-y)

# Additional File 1

## Data Assumptions and Structuring

Height and Weight

Patient’s recorded height and weight during the hospital visit was used. If this data was missing we obtained data from prior inpatient or outpatient records with the assumption that over a 2 year period height and weight remained stable.

Time of intubation (T_i_)

To accurately capture the time of intubation (Ti) the earliest of the following data points were used: an endotracheal intubation procedure note in the electronic medical record; “Start Time” of mechanical ventilation documented in the mechanical ventilation record; time of administration of rapid sequence induction medications (cisatracurium, rocuronium, vecuronium, succinylcholine, etomidate); documentation of ventilator settings by the respiratory therapist in the mechanical ventilation record; nursing documentation of ventilator use in the oxygen delivery method section of the flowsheets; or initiation of continuous infusions of sedative medications (fentanyl, propofol).

Altered Mental Status Prior to Intubation

We used 25 chart items that included mental status data (e.g., Level of Consciousness, Arousal Level, Orientation, and Behavior Monitoring). After reviewing the entries for items at aggregate level, we picked 11 keywords/abbreviations for altered mental status (e.g., AMS, obtunded, confused, and unconscious). Regular Expressions was leveraged to extract different variations of keywords (e.g., comatose and semicomatose) and weed out false positives (e.g., AMS in Tamsulosin). Results were verified using a random sample of 100 outputs. Aggregated results for mental status within 24 hours before the time of intubation is presented in Table 1.

Duration of intubation

The duration of intubation was calculated by identifying the extubation time with the following criteria in the electronic medical record using a step-wise approach. First we searched for the field “time ventilator discontinued” in the mechanical ventilation record. If that data point was unavailable we used the “reason ventilator discontinued” or “ventilator mode off” fields, followed by time of “airway removal note”. In patients where there was evidence of mechanical ventilator records being discontinued, but the above evidence of extubation was missing, the first instance of supplemental oxygen delivery by methods not possible via mechanical ventilation was used. If the patient expired while receiving mechanical ventilation, expiration time was used as time of extubation within 8 hours of last mechanical ventilator record.  Finally, if none of the aforementioned exhaustive criteria were found, we added 4 hours to the last set of ventilator parameters recorded in the mechanical vent record and assumed that as the time of extubation. The duration of invasive mechanical ventilation was defined as the number of days between T_i_ and successful extubation without the need for reintubation within 48 hours, or death.

ICU readmission

Intensive care unit (ICU) readmission was defined as over 48 hours between ICU discharge and ICU readmission and the first visit was used for this analysis. During the surge, hospitals within the system have expanded critical care capacity by creating dedicated COVID ICUs at certain hospitals.

Oxygen Delivery Method, Concentration, and Degree of Hypoxemia:

The FiO_2_ delivered was calculated based on the following formula: for nasal cannula or non-rebreather face mask, each liter of oxygen flow added 0.04 to 0.21 (room air), with a maximum of 6 liters per minute for nasal cannula and 15 liters per minute for non-rebreather mask. In cases where both nasal cannula and non-breather mask was used at the same time, FiO_2_ was assumed to be 1.0. For non-invasive ventilation (NIV), high flow nasal cannula (HFNC), or venturi mask, the documented FiO_2_ was used. Of note, there was limited use of NIV or HFNC because of concerns for airborne transmission of COVID-19 during this particular time. In the instances where the delivery method was not recorded in the electronic medical record, the previous recorded method was presumed to be have been continued, until change in flow rate or delivery method was noted.

To be able to accurately map hypoxemia prior to intubation, we used both arterial blood gas data on partial pressure of oxygen (PaO_2_) and peripherally measured oxygen saturation (SpO_2_). We calculated SpO_2_:FiO_2_ ratios as well as PaO_2_:FiO2 ratios over time for each patient across their entire hospital stay. For separate analyses we converted SpO_2_:FiO_2_ to PaO_2_:FiO_2_ ratios (‘derived P/F’) to obtain an estimated trajectory of PaO_2_ over time (refs). The assumption that derived P/F would have parallel trends compared to ABG derived P/F was tested.

Respiratory System Compliance

We used both static compliance (change in lung volume per unit change in pressure in the *absence of flow)* using the plateau pressure recorded in the electronic medical record, (Tidal Volume/ [Plateau Pressure – Peak End Expiratory Pressure (PEEP]); and dynamic compliance using the Peak Inspiratory Pressure (PIP) (change in lung volume per unit change in pressure in the *presence of flow),*(Tidal Volume/ [PIP – PEEP]) when patients were deeply sedated/paralyzed as described below. We only included values obtained at the time of full patient sedation, which was defined as the administration of intermittent bolus or continuous infusions of paralytics (cisatracurium, rocuronium, vecuronium) and (patient respiratory rate - set respiratory rate) <2 (Figure S6). We made the assumption that patients would not have a significant component of airway resistance for most COVID-19 respiratory failure patients in the early stage of disease (no more than a difference of 5-7 mmHg between PIP and Plateau pressures), and that therefore this added pressure due to flow would have a minimal contribution to overall measured compliance. This assumption was tested by visualizing the difference between static and dynamic compliance seen over time (Figure S3).

Interventions to prevent the worsening of ARDS

We recorded the use of paralytic medications, vasopressor or inotropic medications, lung protective ventilatory settings, steroids, and various experimental treatments that were provided during the surge (azithromycin, vitamin C, hydroxychloroquine, tocilizumab, anakinra, steroids) (Table 2). Proning was defined as a binary variable, and was extracted by string pattern search of items related to the patient position in the EMR. The search discerns positive matches (e,g., “proned”) from the negative ones (e.g., “unproned”).  We created a standardized set and returned tidal volumes for each patient based on predicted body weight, calculated based on sex and height.

**Table S1**. Demographics for total cohort and the group with no reliable compliance data

|  | **Total cohort**  **(n=1,910)** | **Patients with no reliable compliance**  **(n=374)** |
| --- | --- | --- |
| **Age, median (IQ)** | 66.0 (57.0,74.0) | 69.0 (61.0,76.0) |
| **Female**n (%) | 633 (32.0) | 143 (33.6%) |
| **Race** | | |
| **African American/Black** n (%) | 348 (18.2%) | 64 (17.1%) |
| **Asian** n (%) | 182 (9.5%) | 30 (8.0%) |
| **Other/Multiracial** n (%) | 578 (30.3%) | 97 (25.9%) |
| **White** n (%) | 699 (36.6%) | 162 (43.3%) |
| **Unknown** n (%) | 103 (5.4%) | 21 (5.6%) |
| **Comorbidities** | | |
| **Charlson Index**mean (SD) | 5.1 (3.3) | 5.8 (3.4) |
| **MEWS on admission**mean (SD) | 4.1 (1.9) | 4.2 (1.9) |
| **BMI on admission**mean (SD) | 31.6 (34.8) | 34.1 (77.9) |
| **Chronic Lung Disease**n (%) | 138 (7.2%) | 34 (9.1%) |
| **Diabetes**n (%) | 861 (45.1%) | 195 (52.1%) |
| **HTN**n (%) | 1,252 (65.5%) | 251 (67.1%) |
| **CHF**n (%) | 155 (8.1%) | 40 (10.7%) |
| **CAD**n (%) | 268 (14.0%) | 64 (17.1%) |
| **CKD**n (%) | 291 (15.2%) | 65 (17.4%) |
| **ESRD**n (%) | 104 (5.4%) | 23 (6.1%) |
| **Positive Smoking**n (%) | 239 (12.5%) | 42 (11.2%) |
| **Malignancy**n (%) | 193 (10.1%) | 47 (12.6%) |
| **Altered Mental Status 24h before intubation** n (%) | 507 (26.5%) | 119 (31.8%) |
| **BMI Categories**n (%) | | |
| **BMI <18 underweight** | 19 (1.0%) | 10 (2.7%) |
| **BMI 18 – <30 normal–overweight** | 919 (48.1%) | 186 (49.7%) |
| **BMI 30 – <40 obese** | 666 (34.9%) | 116 (31.0%) |
| **BMI >=40 extremely obese** | 306 (16.0%) | 62 (16.6%) |

Table S2. Interventions for total cohort and the group with no reliable compliance data

|  | **Total cohort**  **(n=1,910)** | **Patients with no reliable compliance**  **(n=374)** |
| --- | --- | --- |
| **Steroids**n (%) | 1,549 (81.1%) | 277 (74.1%) |
| **Hydroxychloroquine**n (%) | 1,677 (87.8%) | 301 (80.5%) |
| **Azithromycin**n (%) | 1,179 (61.7%) | 221 (59.1%) |
| **IL–1 or IL–6 inhibitor**n (%) | 570 (29.8%) | 96 (25.7%) |
| **Remdesivir**n (%) | 12 (0.6%) | 1 (0.3%) |
| **Convalescent plasma**n (%) | 125 (6.5%) | 16 (4.3%) |
| **Proning**  n (%) | 902 (47.2%) | 136 (36.4%) |
| **Proning before intubation**n (%) | 324 (17.0%) | 43 (11.5%) |
| **Paralytics**n (%) | 906 (47.4%) | 93 (24.9%) |
| **Vasopressors (y/n) in first 48hrs post intubation** n (%) | 1,468 (76.9%) | 241 (64.4%) |
| **Inotropes (y/n) at any time count**n (%) | 75 (3.9%) | 10 (2.7%) |
| **Median hours from hospital presentation to intubation (IQR)** | 48.8 (6.8,124.4) | 37.1 (2.7,121.1) |
| **CT pulmonary angiogram (CTPA) performed within [-1 to 7 days] of intubation** n (%) | 67 (3.5%) | 19 (5.1%) |
| **Pulmonary embolus identified on CT** n (% of patients with CTPA) | 9 (13.4%) | 3 (15.8%) |
|  | **Pre–intubation O2 supplementation**n (%) | |
| **NRB** | 1,440 (75.4%) | 254 (67.9%) |
| **NRB+NC** | 45 (2.4%) | 4 (1.1%) |
| **NC** | 115 (6.0%) | 35 (9.4%) |
| **HFNC** | 41 (2.1%) | 9 (2.4%) |
| **NIV (BiPAP/CPAP)** | 60 (3.1%) | 8 (2.1%) |
| **Venturi** | 19 (1.0%) | 3 (0.8%) |
| **Other** | 190 (9.9%) | 61 (16.3%) |

Table S3. Oxygenation trends and duration of ventilation by compliance group for total cohort and the group with no reliable compliance data

|  | **Total cohort**  **(n=1,910)** | **Patients with no reliable compliance**  **(n=374)** |
| --- | --- | --- |
| **P/F *derived* pre intubation (12hr mean)**  Mean (SD)  Median (IQR)  n | 96.84 (87.63)  60.49 (53.15,104.82)  1764 | 112.01 (103.64)  63.43 (56.08,132.52)  327 |
| **P/F from *ABG* pre intubation (12hr mean)**  Mean (SD)  Median (IQR)  n | 109.21 (90.80)  76.54 (61.73,112.91)  546 | 119.55 (108.38)  82.72 (63.33,114.00)  93 |
| **P/F *derived* post intubation, (12hr mean)**  Mean (SD)  Median (IQR)  n | 68.07 (40.52)  53.82 (40.85,81.82)  1905 | 71.70 (48.10)  54.24 (39.88,87.04)  372 |
| **P/F from *ABG* post intubation (12hr mean)**  Mean (SD)  Median (IQR)  n | 154.50 (78.17)  136.40 (95.71,195.31)  1823 | 169.38 (90.96)  144.00 (98.00,223.56)  347 |
| **First ABG P/F post intubation (within 4 hours after Ti),**  Mean (SD)  Median (IQR)  n | 144.61 (86.70)  119.00 (84.00,183.00)  1370 | 152.06 (90.16)  120.00 (86.06,201.78)  254 |
| **P/F gradient *derived* 24 hrs pre intubation (derived P/F)**  Mean (SD)  Median (IQR)  n | -174.90 (1615.73)  -7.00 (-102.76,1.56)  1506 | -97.61 (526.33)  -4.95 (-102.15,3.96)  270 |
| **Lactate pre intubation (24hr mean)**  Mean (SD)  Median (IQR)  n | 1.82 (2.46)  1.30 (0.95,1.81)  228 | 1.36 (1.36)  1.09 (0.80,1.49)  28 |
| **Lactate post intubation (24hr mean)**  Mean (SD)  Median (IQR)  n | 1.66 (1.55)  1.30 (0.90,1.80)  210 | 1.52 (1.04)  1.15 (0.83,1.86)  26 |
| **Number of hours with FiO2 ≥ 60% pre intubation**  Mean (SD)  Median (IQR)  n | 61.27 (97.12)  20.98 (1.85,77.96)  1812 | 46.69 (79.43)  11.77 (0.50,64.03)  339 |
| **Proportion of time on FiO2 ≥ 60% pre–intubation**  Mean (SD)  Median (IQR)  n | 61.4% (36.8%)  71.9% (27.6%,98.2%)  1709 | 56.4% (38.2%)  63.7% (17.8%,98.7%)  306 |
| **Proportion of time on FiO2 ≥ 60% post–intubation**  Mean (SD)  Median (IQR)  n | 53.6% (35.0%)  51.1% (19.4%,91.4%)  1910 | 59.0% (36.0%)  58.9% (24.1%,100.0%)  374 |
| **Oxygenation Index**  Mean (SD)  Median (IQR)  n | 11.19 (6.29)  10.08 (6.72,14.16)  1799 | 10.73 (7.71)  9.09 (5.64,13.92)  330 |
| **PFP value**  Mean (SD)  Median (IQR)  n | 151.45 (113.43)  120.37 (83.74,178.75)  1894 | 179.17 (146.79)  133.45 (79.47,212.88)  364 |
| **pH within –24 to +4 hours from T_i_**  Mean (SD)  Median (IQR)  n | 7.30 (0.13)  7.32 (7.22,7.39)  1365 | 7.29 (0.15)  7.33 (7.19,7.40)  246 |
| **PaCo2 within –24 to +4 hours from T_i_**Mean (SD)  Median (IQR)  n | 50.23 (17.19)  46.00 (38.00,58.50)  1489 | 47.58 (16.19)  43.00 (36.00,55.00)  288 |

Table S4. Mechanical ventilator obtained parameters (lung mechanics and ventilator settings) for total cohort and the group with no reliable compliance data

|  | **Total cohort**  **(n=1,910)** | **Patients with no reliable compliance**  **(n=374)** |
| --- | --- | --- |
| **Mean PEEP (cm H2O) within 24 hrs of intubation**  Mean (SD)  Median (IQR)  n | 12.42 (3.88)  12.14 (10.00,15.00)  1910 | 11.79 (4.31)  11.34 (8.93,15.00)  374 |
| **Mean Peak pressure (cm H2O) within 24 hrs of intubation**  Mean (SD)  Median (IQR)  n | 32.48 (6.90)  32.00 (28.00,36.42)  1907 | 30.93 (7.98)  30.00 (25.53,35.00)  371 |
| **Ventilation duration (days)**  Mean (SD)  Median (IQR)  n | 14.39 (16.34)  9.02 (3.82,18.96)  1910 | 10.84 (14.97)  5.30 (1.55,14.19)  374 |
| **Ventilation duration (days) among those who survived,**  Mean (SD)  Median (IQR)  n | 19.83 (20.18)  12.00 (4.94,29.25)  567 | 19.17 (19.75)  10.98 (4.19,29.59)  88 |
| **Ventilation duration (days) among those who died**  Mean (SD)  Median (IQR)  n | 12.05 (13.77)  7.95 (3.35,16.49)  1343 | 8.27 (12.09)  4.35 (1.04,10.96)  286 |
| **Vt cc/Kg of IBW** **within 24 hrs of intubation**  Mean (SD)  Median (IQR)  n | 6.78 (1.22)  6.64 (6.05,7.36)  1785 | 6.82 (1.42)  6.68 (6.02,7.54)  344 |
| **Set respiratory rate (per minute)** **within 24 hrs of intubation**  Mean (SD)  Median (IQR)  n | 23.70 (5.26)  24.00 (20.00,27.73)  1910 | 21.50 (5.32)  20.00 (18.00,25.43)  374 |
| **Total respiratory rate within 24 hrs of intubation**  Mean (SD)  Median (IQR)  n | 25.70 (4.79)  25.67 (22.25,29.19)  1890 | 25.61 (4.96)  25.67 (22.00,29.00)  354 |
| **Plateau pressure (cm H2O) within 24 hrs of intubation**  Mean (SD)  Median (IQR)  n | 28.04 (6.72)  27.50 (24.00,32.00)  1251 | 26.33 (7.17)  25.00 (22.33,30.00)  202 |
| **Driving pressure (cm H20) within 24 hrs of intubation**  Mean (SD)  Median (IQR)  n | 16.00 (6.45)  15.00 (12.00,19.00)  1244 | 15.06 (6.42)  14.00 (11.33,17.54)  200 |

Table S5. Hospital mortality and discharge location for total cohort and the group with no reliable compliance data

|  | **Total cohort**  **(n=1,910)** | **Patients with no reliable compliance**  **(n=374)** |
| --- | --- | --- |
| **Deceased**% (n) | 70.3% (1,343) | 76.5% (286) |
| **Survived**% (n) | 29.7% (567) | 23.5% (88) |
| **Discharged while on mechanical ventilator,**% of the survivors (n) | 14.1% (80)19.3% (17) | 14.1% (80)19.3% (17) |
| **Discharged home,**% of the survivors (n) | 54.3% (308)40.9% (36) | 54.3% (308)40.9% (36) |
| **Discharged to another facility including acute care and longer-term rehabilitation,**% of the survivors (n) | 45.5% (258) | 59.1% (52) |

**Figure S1.** Trend calculation method for P/F ratio. First, we use exponential smoothing to reduce the noise in data (red diamond points). We then find OLS regression line passing through the smoothed points. Trend is the slope of the line (i.e., 74.29).


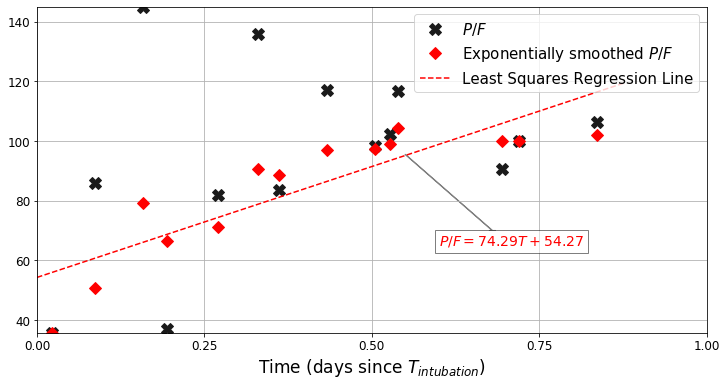


**Figure S2.** Comparison of derived P/F and ABG based P/F ratios.  **a.** Trends appear similar over time in very low and low-normal categories of lung compliance, and with ABG P/F (top), derived P/F from SpO2 (middle) and combining both methods with preference for ABG P/F (bottom). The ABG derived P/F has large variation due to fewer datapoints. The very low compliance category consistently has lower P/F ratio which is more pronounced pre–intubation.  **b.** P/F ratios in the first 24 hours of intubation are higher with ABG P/F (top) compared to derived ABG (bottom). Derived P/F underestimates the ratio (due to lower derived PaO2) due to oxygen dissociation curve where there is a greater range of PaO2 for a given SpO2, and the fixed upper limit of SpO2 at 100%.


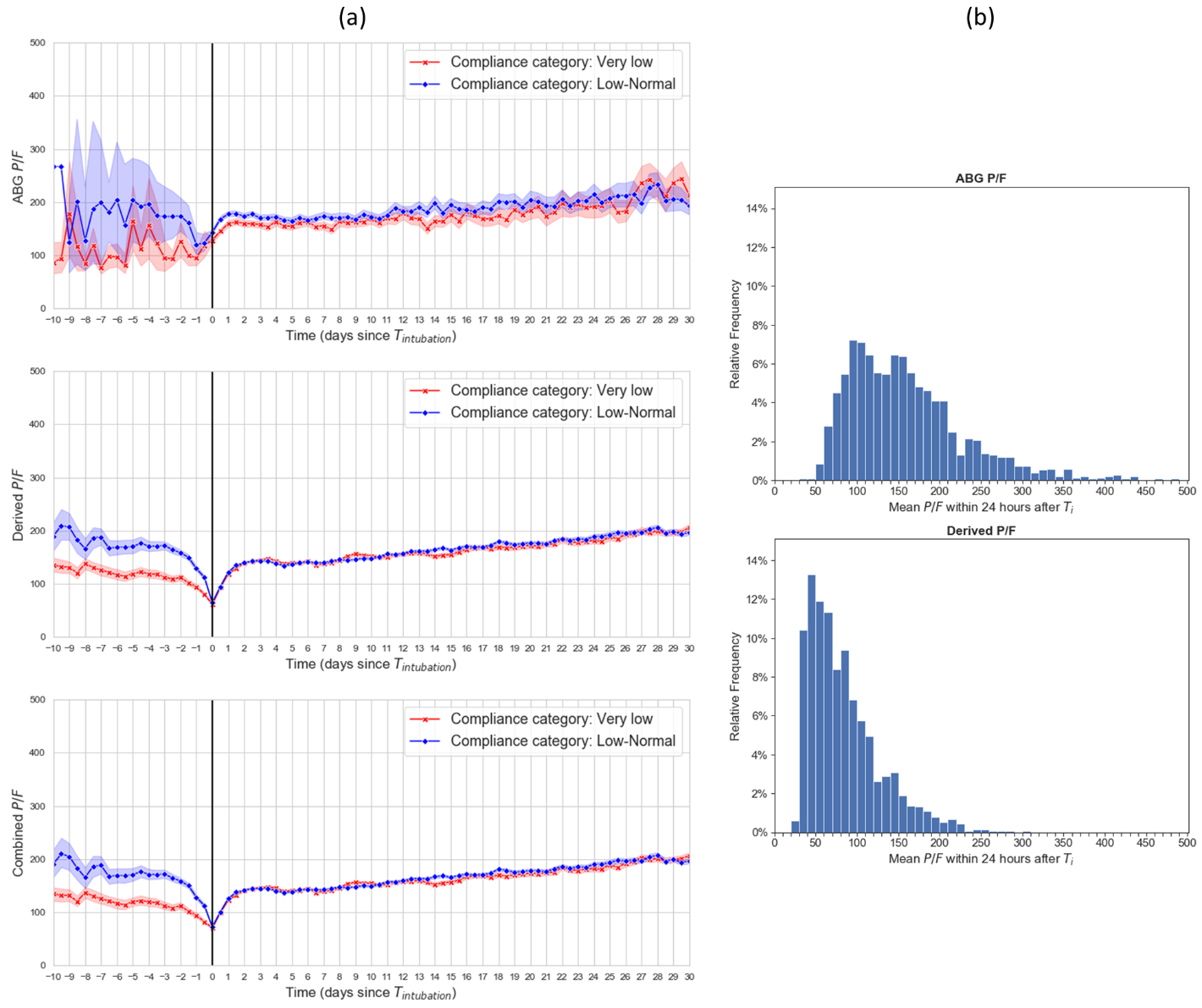


**Figure S3**. Static versus Dynamic Compliance over time to test the assumption that the dynamic compliance would be continuously lower than static with a relatively fixed difference over time, and regardless of compliance category.


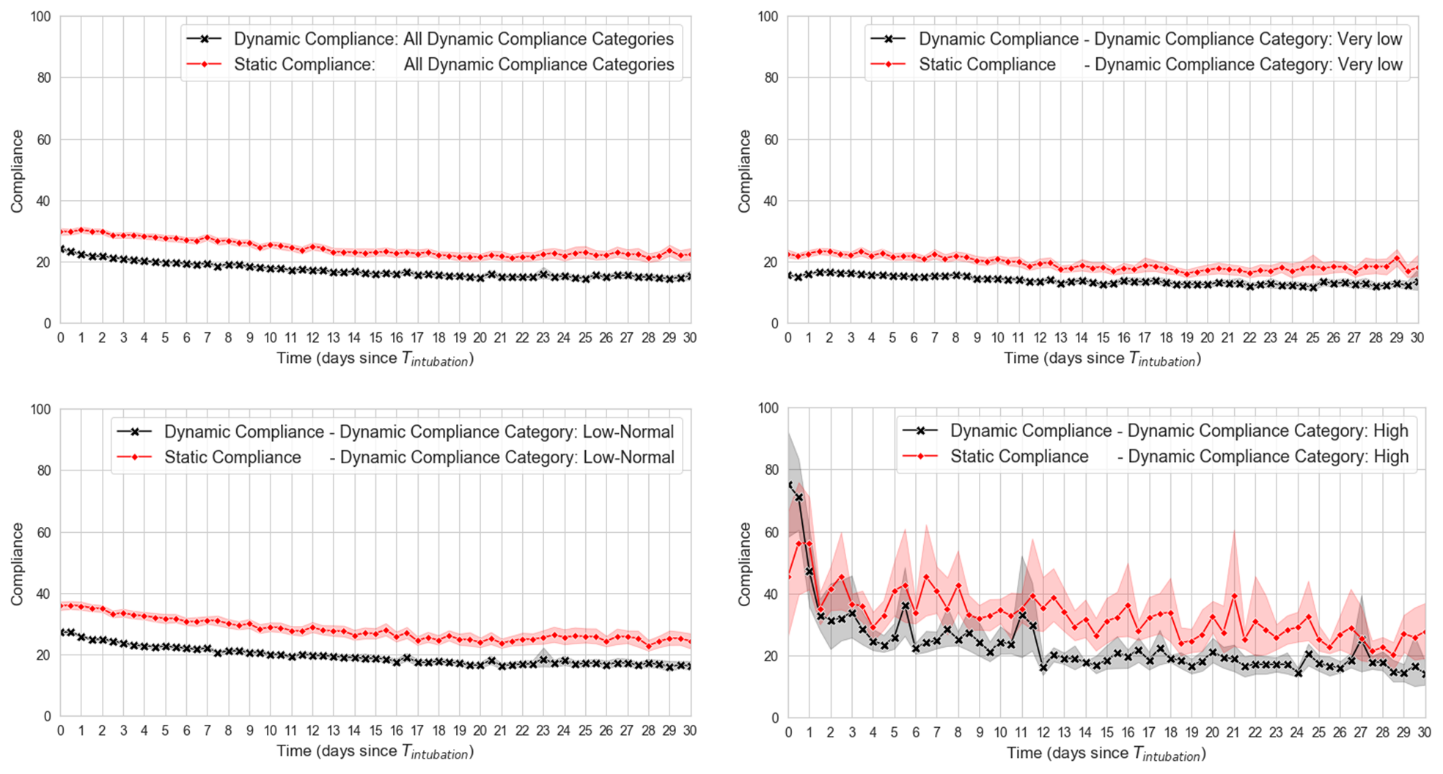


**Figure S4.** Comparison of Dynamic Compliance change over time (shaded areas represent 95% Confidence Intervals). **a.** Comparison of all three categories. **b.** comparison between very low and low-normal categories. A steeper trajectory is seen for the high and low–normal categories over time. Large variability seen in the high compliance group secondary to small sample size.

**(a)**


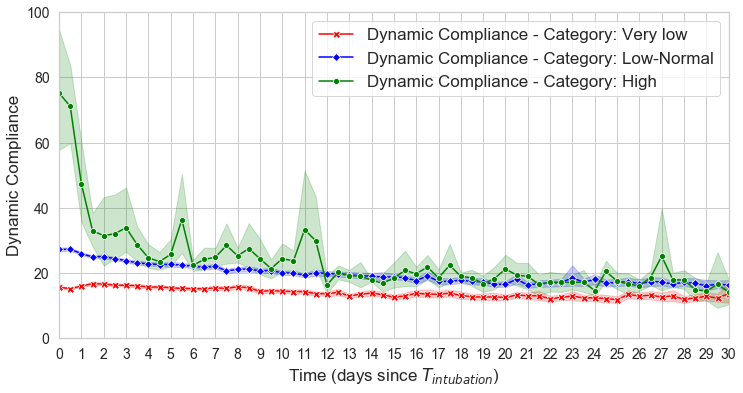


**(b)**


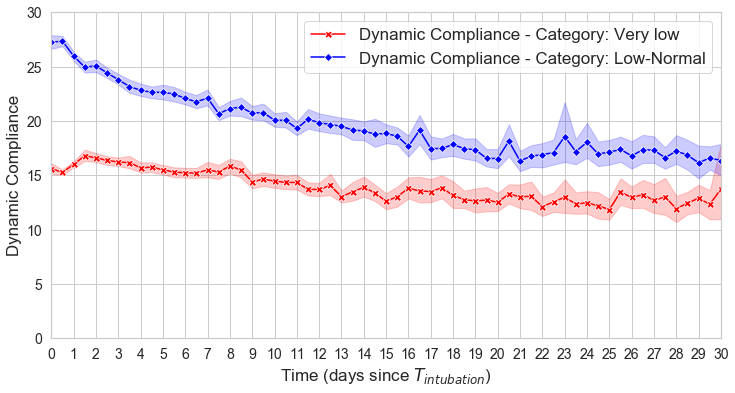


**Figure S5.** Distribution of PaO2 and SpO2 **a.** 24 hours before intubation (on left hand side) and after (on the right hand side) intubation (Ti) **b.** mean SpO2 and PaO2 association **c.** trend in SpO2 and PaO2 over the entire hospital course.

**(a)** 

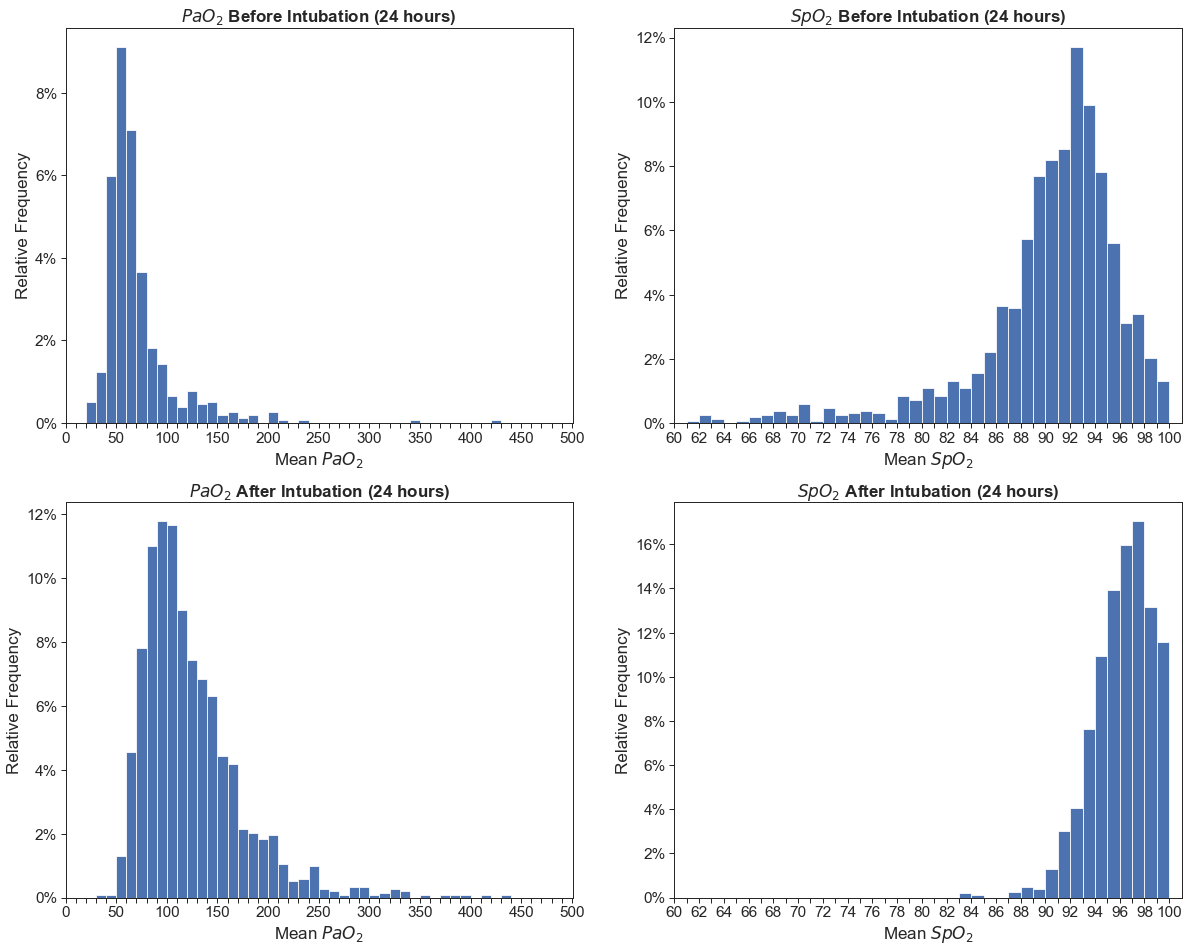


**(b)**


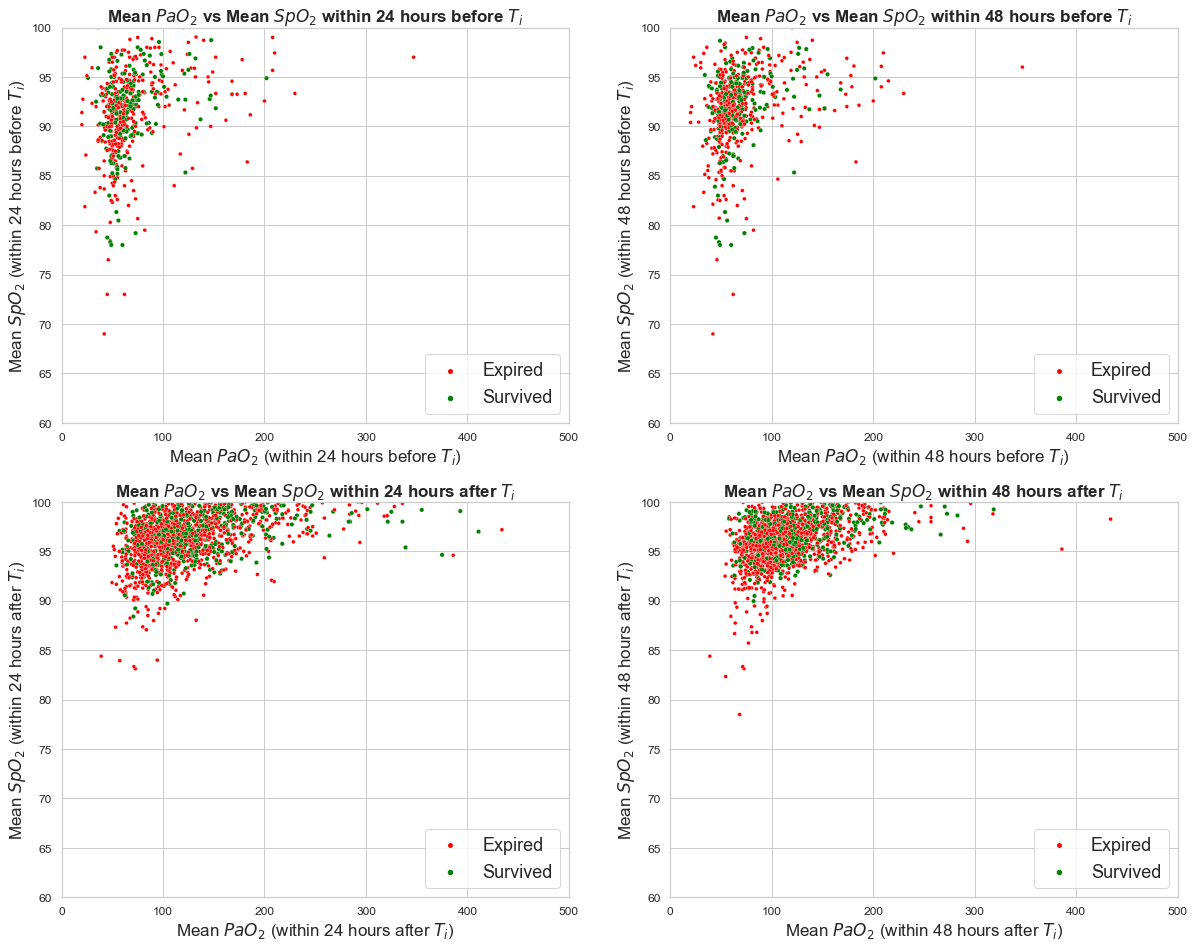


**(c)**


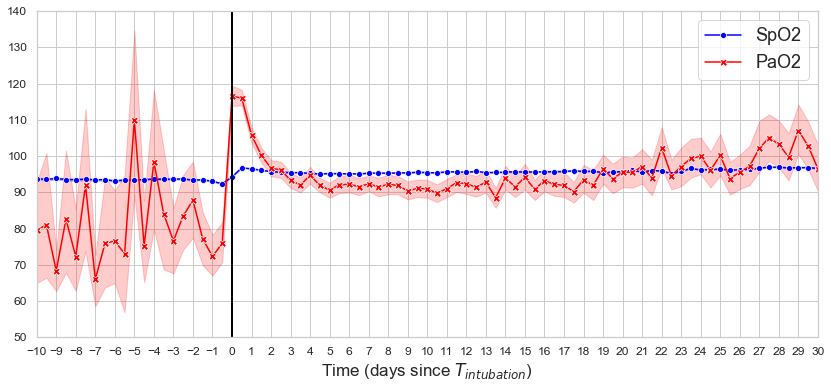


**Figure S6**.  Visualization of assumptions made to detect valid respiratory system compliance measurements using a sample patient. The top figure shows all available measurements which could be used to calculate compliance, with green dots indicating when the measurements were deemed to be acceptable by the algorithm based on either being on a continuous paralytic agent or sedative with difference in set versus measured respiratory rate less than 2 breaths per minute (ΔRR <2).  The bottom figure shows the ΔRR which corresponds to each timepoint of valid compliance measurement. A measurement is acceptable (green dots in the top figure) if the patient was deeply sedated and or paralyzed (green lines in the bottom figure), and were breathing at the rate set by the ventilator (ΔRR <2 in the bottom figure).


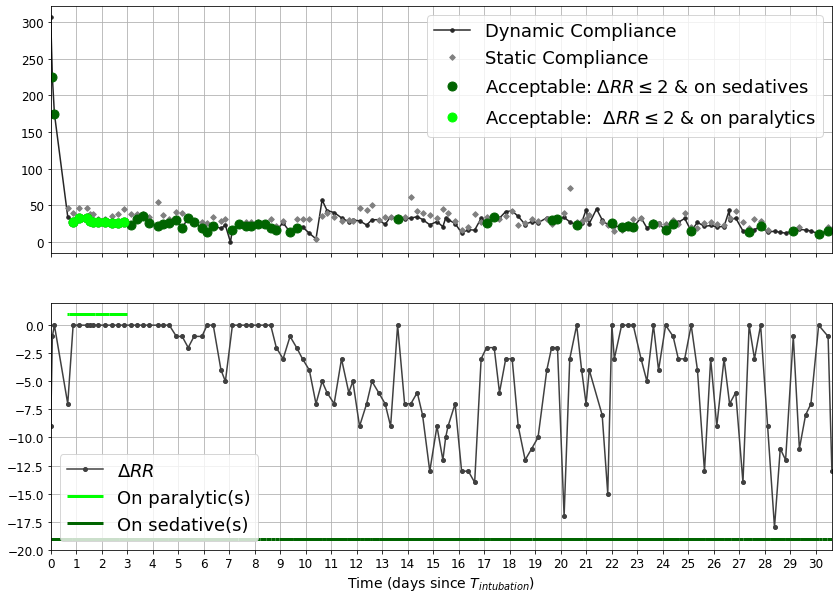


**Figure S7**.  PFP trend over time for the entire cohort (PFP=[P/(F × PEEP)] × 10, calculated using ABG PaO2).

 
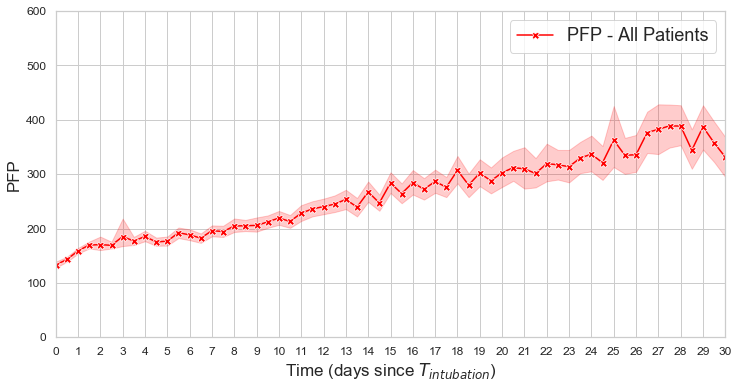

Supplement: Supplementary file 1 — Additional file 1. Data Assumptions and Structuring. [file 12890_2021_1732_MOESM1_ESM.docx]
